# Genome-Wide Identification of Calcium-Dependent Protein Kinases in *Chlamydomonas reinhardtii* and Functional Analyses in Nitrogen Deficiency-Induced Oil Accumulation

**DOI:** 10.3389/fpls.2019.01147

**Published:** 2019-10-22

**Authors:** Yajun Li, Xiaowen Fei, Haofu Dai, Jiangyue Li, Weiju Zhu, Xiaodong Deng

**Affiliations:** ^1^Hainan Provincial Key Laboratory for Functional Components Research and Utilization of Marine Bio-resources, Institute of Tropical Bioscience and Biotechnology, Hainan Academy of Tropical Agricultural Resource, Chinese Academy of Tropical Agricultural Sciences, Haikou, China; ^2^Biochemistry and Molecular Biology Department, Hainan Medical College, Haikou, China

**Keywords:** *Chlamydomonas reinhardtii*, Ca^2+^-dependent protein kinases, expression pattern, gene function, nitrogen deficiency, oil accumulation

## Abstract

Calcium-dependent protein kinases (CDPKs) are recognized as important calcium (Ca^2+^) sensors in signal transduction and play multiple roles in plant growth and developmental processes, as well as in response to various environmental stresses. However, little information is available about the CDPK family in the green microalga *Chlamydomonas reinhardtii*. In this study, 15 *CrCDPK* genes were identified in *C. reinhardtii* genome, and their functions in nitrogen (N) deficiency-induced oil accumulation were analyzed. Our results showed that all CrCDPK proteins harbored the typical elongation factor (EF)-hand Ca^2+^-binding and protein kinase domains. Phylogenetic analysis revealed that these CrCDPKs were clustered into one group together with a subclade of several CPKs from *Arabidopsis* and rice, clearly separating from the remaining AtCPKs and OsCPKs. These genes were located in 10 chromosomes and one scaffold of *C. reinhardtii* and contained 6–17 exons. RNA sequencing and quantitative reverse transcription (qRT)-PCR assays indicated that most of these *CrCDPK*s were significantly induced by N deficiency and salt stress. Lanthanum chloride (LaCl_3_), a plasma membrane Ca^2+^ channel blocker, limited oil accumulation in *C. reinhardtii* under N-deficient conditions, suggesting that Ca^2+^ was involved in N deficiency-induced oil accumulation. Furthermore, RNA interference (RNAi) silencing analyses demonstrated that six *CrCDPK*s played positive roles and three *CrCDPK*s played negative roles in N deficiency-induced oil accumulation in *C. reinhardtii*.

## Introduction

Calcium ion (Ca^2+^), a ubiquitous second messenger in cell signaling, plays essential roles in many physiological processes, such as plant growth, development, and abiotic and biotic stresses (Hetherington and Brownlee, 2004). Various extracellular stimuli can cause transient and minor changes in cytosolic Ca^2+^ concentration, which can be first sensed and decoded by several Ca^2+^ sensors, triggering appropriate downstream responses in plant cells (Lenzoni et al., 2018). In general, three classes of Ca^2+^ sensors, namely, calcineurin B-like (CBL), calmodulin (CaM) and CaM-like proteins, and Ca^2+^-dependent protein kinases (CDPKs or CPKs), have been identified (Boudsocq and Sheen, 2013). However, only CDPKs can directly transduce Ca^2+^ signal into downstream phosphorylation cascades by a single gene product, which arise from their special structures (Hu et al., 2016b). The CDPK family harbors four functional domains, namely, the variable N-terminal domain, highly conserved Ser/Thr kinase domain, autoinhibitory junction domain, and CaM-like domain. The N-terminal domain contains potential myristoylation or palmitoylation sites for membrane association, and the Ser/Thr kinase domain is required for substrate phosphorylation. The autoinhibitory junction domain modulates the kinase activity of CDPKs *via* a pseudosubstrate mechanism, and the CaM-like domain contains four elongation factor (EF)-hand motifs for binding to Ca^2+^ (Liese and Romeis, 2013; Schulz et al., 2013; Liu et al., 2018). Ca^2+^ binding to EF-hands induces a conformational change in CDPK, resulting in the refolding and exposure of the active site of the kinase domain and thus activating the CDPK to phosphorylate an array of substrates.

A large number of CDPKs have been widely found in plants, green algae, and certain protozoans but not in bacteria, fungi, yeast, and animals (Batistič and Kudla, 2012; Hamel et al., 2014). For instance, 34 and 31 CDPKs have been identified in *Arabidopsis* and rice genomes, respectively (Hrabak et al., 2003; Ray et al., 2007). In addition, there are 20 CDPKs in wheat genome (Li et al., 2008), 23 in potato (Gromadka et al., 2018), 27 in cassava (Hu et al., 2016a), 29 in tomato (Hu et al., 2016b), 30 in poplar (Zuo et al., 2013), 40 in maize (Kong et al., 2013), and 41 in cotton (Liu et al., 2014). The biological functions of CDPKs, including pollen tube development (AtCPK2/AtCPK11/AtCPK17/AtCPK20/AtCPK24/AtCPK34) (Gutermuth et al., 2013; Zhao et al., 2013), response to environmental stresses (AtCPK1/AtCPK3/AtCPK4/AtCPK5/AtCPK6/AtCPK8/AtCPK10/AtCPK11/AtCPK13/AtCPK21) (Choi et al., 2005; Mori et al., 2006; Zhu et al., 2007; Boudsocq et al., 2010; Franz et al., 2011; Zhou et al., 2015), and plant growth regulation (AtCPK12/AtCPK28) (Zhao et al., 2011; Matschi et al., 2013), have been well characterized in *Arabidopsis*. Several rice CDPKs have been demonstrated to respond to cold (OsCPK7/OsCPK13/OsCPK17/OsCPK24) (Saijo et al., 2000; Almadanim et al., 2018; Liu et al., 2018), drought, and salt stresses (OsCPK4/OsCPK9/OsCPK12/OsCPK13/OsCPK21) (Asano et al., 2011; Asano et al., 2012; Campo et al., 2014; Wei et al., 2014). By contrast, information about the roles of CDPKs derived from other plant species is still scarce, apart from a few CDPKs that are involved in tolerance to abiotic stresses (ZmCPK1, VaCPK20, BnaCPK2, and StCDPK4/5) (Kobayashi et al., 2007; Dubrovina et al., 2015; Weckwerth et al., 2015; Wang et al., 2018b).

Microalgae are a large and diverse group of photosynthetic eukaryotes with a simple cellular structure, which range from unicellular to multicellular forms. In general, the lipid metabolic pathways in microalgae were inferred from those of plants wherein the lipid synthesis have been well characterized. However, the recent studies have shown that there were many differences between algal species and higher plants. For instance, cells usually contain betaine lipid diacylglyceryl-(*N*,*N*,*N*-trimethyl)homoserine (DGTS) instead of phosphatidylcholine (PtdCho) in *Chlamydomonas reinhardtii* (Liu and Benning, 2013). With the development of next-generation sequencing technology, more and more algal genes involved in lipid metabolism were identified, and the gene functions were annotated to different extent in microalgae. The lipid biochemistry of eukaryotic algae has been reviewed by Li-Beisson et al. (2019). Stress conditions, especially nitrogen (N) deficiency, can induce oil droplet biosynthesis, mainly in the form of triacylglycerol (TAG), which accumulates in cells to generate lipid droplets that store carbon and energy in many microalgae species, such as *C. reinhardtii* (Hu et al., 2008). During N deprivation, massive metabolic alterations were observed in *C. reinhardtii*. Wase et al. (2014) reported that, compared with non-stressed cultures, metabolites and proteins involved in citrate [tricarboxylic acid (TCA)] cycles, glycolysis, and starch and lipid metabolism were elevated, and the enzymes of the glyoxylate cycle, the Calvin cycle, and photosynthesis were reduced in response to N starvation. These metabolic changes play important roles in the supply of carbon skeletons and energy for lipid storage under N-deficient conditions. Moreover, citrate accumulated during nitrogen deprivation, which may be exported for use in fatty acid (FA) synthesis. Similar results were obtained by Park et al. (2015). Once N deficiency-induced TAG synthesis was initiated in the cells, carbon metabolism shifted from a gluconeogenic state to a glycolytic state. In addition, the transcriptional levels of genes related to pyruvate and acetyl coenzyme A (acetyl-CoA) synthesis steadily increased during N starvation. Pyruvate and acetyl-CoA are two central intermediates linking glycolysis and TCA cycles to lipid metabolism, respectively.

N deficiency-induced microalgal oil accumulation always associated with reduced biomass yield, which was a large obstacle for algal-based biodiesel production. Currently, an alternative strategy has been developed to select small-molecule chemical inducers to trigger TAG accumulation without terminating cell growth. A total of 243 compounds that clustered into five distinct structural scaffolds were effective in multiple algal species including *Chlamydomonas*, *Chlorella sorokiniana*, *Chlorella vulgaris*, and *Tetrachlorella alternans* (Wase et al., 2017), and 34 molecules were also identified to act as inducers of TAG accumulation in *Phaeodactylum tricornutum* (Conte et al., 2018). Possible targets for these compounds were distributed in various metabolic steps of carbon and lipid metabolism such as lipase inhibition, acyltransferase activity modulation, and channeling of acetyl-CoA to FA biosynthesis (Wase et al., 2018).

Even though many efforts have been made to exploit lipid metabolism in microalgae, its molecular mechanisms remain unclear and controversial. Most evidence shows that CDPKs act as signaling hubs in plant stress signaling and development (Schulz et al., 2013), suggesting their contribution to stress-related TAG accumulation in microalgae. In this study, we identified 15 *CDPK* genes on the basis of a systematic genome-wide analysis in *C. reinhardtii* and analyzed their protein motifs, phylogenetic relationship, chromosome localization, gene structure, expression profiles under various abiotic stresses, and the functions of *CrCDPK*s in N deficiency-induced oil accumulation. RNAi silencing of *CrCDPK*s resulted in a significant decrease or increase in the oil content of *C. reinhardtii* under N deficiency.

## Materials and Methods

### Identification of *CDPK* Genes in *C. reinhardtii*

The BLASTP program at an expectation value (E) of 10^−10^ was performed in Phytozome V11.0 *C. reinhardtii* v5.5 database (https://phytozome.jgi.doe.gov/) by using the published CDPK proteins (*Arabidopsis thaliana* and *Oryza sativa*) as query sequences (**Table S1**). In addition, another way to identify the *CDPK* genes was searching the database using the keyword “calcium-dependent protein kinase kinase.” Only non-redundant full-length sequences containing EF-hand motifs were considered. Conserved protein motifs were predicted using ScanProsite (http://prosite.expasy.org/) and SMART (http://smart.embl-heidelberg.de/). The protein domains were visualized by the Interactive Tree of Life (iTOL) (http://itol2.embl.de/). The molecular weights and isoelectric points of proteins were predicted using ExPASy (http://web.expasy.org/compute_pi/). Myristoylation sites were predicted by NMT—the MYR predictor script (http://mendel.imp.ac.at/myristate/SUPLpredictor.htm).

### Phylogenetic Analysis, Chromosomal Locations, and Gene Structure of *CrCDPK*s

Multiple sequence alignments were performed with Clustal X 2.0, and a phylogenetic tree was constructed on the basis of the neighbor-joining method using MEGA 6.0 software with 1,000 bootstrap replicates. The chromosomal locations of 15 *CrCDPK* genes were drafted using MapInspect 1.0. The structures of *CrCDPK* genes were depicted by using the Gene Structure Display Server (GSDS) (http://gsds.cbi.pku.edu.cn/)

### Algal Strain, Growth Condition, and Stress Treatments

*C. reinhardtii* CC425 (cw15 arg2) was purchased from the *Chlamydomonas* Genetic Center of Duke University. For RNA-Seq analysis, the wild-type CC425 was first grown in Tris–acetate–phosphate (TAP) medium for 3 days to reach a growth plateau and then collected by centrifugation (3,000 *g*). After being washed twice with sterile water, the algal cells were inoculated in high salt minimal (HSM) medium that is deficient in N, phosphorus (P), and iron (Fe) and contains 100 mM of NaCl (salt stress). They were placed in an incubator shaker maintained at 230 rpm and 24 °C and exposed to continuous illumination at a light intensity of 150 μmol·m^−2^·s^−1^. The cultured cells that reached a growth plateau were inoculated in normal HSM medium and were divided into two groups: one group was used as control, and the other group was placed under a temperature of 16 °C (cold stress). Samples were collected after 24 h for RNA-Seq analysis. N deficiency-treated algal cells were also collected after 0, 2, 12, 24, 48, and 72 h for quantitative reverse transcription (qRT)-PCR.

### RNA-Seq and qRT-PCR

RNA-Seq analysis was performed on an Illumina HiSeq 2000 platform in GENE DENOVO, Guangzhou, China (http://www.genedenovo.com), as described by Li et al. (2017). Raw reads were first filtered by removing the adapter sequences, low-quality sequences, and reads with more than 10% unknown nucleotides. The obtained clean reads were mapped to the Ribosomal Database Project with Bowtie (Langmead et al., 2009), and the reads that belonged to ribosomal DNA (rRNA) were removed. The remaining clean reads were mapped to the reference genome of *C. reinhardtii* by TopHat2 (Kim et al., 2013). The transcriptome was assembled with Cufflinks (Trapnell et al., 2010), and Cuffmerge was used to merge the assembly of two replicate samples. The gene expression level was calculated and normalized by using the FPKM (fragments per kilobase of transcript per million mapped reads) method. The edgeR package (http://www.r-project.org/) was used to identify differentially expressed genes across samples. The false discovery rate (FDR) was used to determine the *p*-value threshold in multiple tests. Genes with FDR ≤0.05 and an absolute value of log_2_ change fold ≥1 were regarded as significant differentially expressed genes.

The fold change value of each stress treatment compared with the control was normalized to complete hierarchical clustering analysis, and the heatmap was drawn by using the R3.5.1 software. For qRT-PCR, total RNA was extracted using TRIzol Reagent (Takara, Japan) and subjected to RNase-free DNase (RQ1, Promega, USA) for digestion and purification. qRT-PCR analyses were performed as described by Li et al. (2017). The gene-specific primers of qRT-PCR are listed in **Table S2**. The experiment was repeated three times, and statistical analysis was performed by using SPSS software with independent-samples *t*-test.

### Lanthanum Chloride (LaCl_3_) Treatment, Cell Viability Assay, and Measurement of Chlorophyll and Oil Contents

LaCl_3_ was purchased from Sigma (St. Louis, MO). *C. reinhardtii* CC425 cells grown on TAP agar plate were inoculated into 100-ml Erlenmeyer flasks containing 50 ml of TAP media for 3 days to reach a growth plateau and were collected by centrifugation at 3,000 *g*. After being washed twice with sterile water, the cultured cells were inoculated into P- and N-and-P-deficient HSM media with various concentrations of LaCl_3_ (0, 1, 5, 10, 25, 50, 75, 100, 150, 200, 500, and 1,000 μM). Moreover, 0 μM of LaCl_3_-treated cells was used as control. All cultures were maintained in an incubator shaker at 230 rpm and 24 °C and exposed to continuous illumination at a light intensity of 150 μmol·m^−2^s^−1^. Samples for cell viability assay and measurement of chlorophyll and oil contents were collected after 24, 48, and 72 h. For cell viability assay, the algal cells were directly stained with 0.01% (w/v) fluorescein diacetate (FDA) (final concentration) for 2 min, and flow cytometry using CyFlow Cube 6 was employed to count cells. A total of 10^4^ cells were counted per sample. Cellular viability was shown as the percentages of FDA-stained live cells to total 10^4^ cell numbers.

The Nile Red fluorescence method was performed to determine the oil content as described by Li et al. (2012a). The algal cells were directly stained with 0.1 mg/ml (final concentration) Nile Red (dissolved in DMSO) for 10 min, and fluorescence was measured on a GloMax-Multi Detection System (Promega, USA) with excitation and emission wavelengths of 470 and 570 nm, respectively. The fluorescence value was calculated by using the equation FD (470/570) = (A2 − A1), where A2 and A1 are the fluorescence values of algal cells after and before staining with Nile Red, respectively. To establish the relationship of fluorescence values of samples with their neutral lipid contents, a standard curve was drawn by preparing different concentrations of Triolein (Sigma, USA) and detecting their fluorescence value after staining with Nile Red. The lipid content of algal cells was calculated using the following formula: lipid content (μg/10^6^ cell) = [0.0004 × FD (470/570) − 0.0038] × 50/cell density. Chlorophyll content was measured as described by Li et al. (2012b). Statistical analysis was performed using the SPSS software by one-way ANOVA and Duncan’s multiple range tests, and the experiment was repeated three times.

### Construction of *CrCDPK*-RNAi Vectors and Transformation of *C. reinhardtii*

To construct the RNAi vectors, a fragment of the *C. reinhardtii* 18S gene was amplified with primers 5′-CGAACTTCTGCGAAAGCAT-3′ and 5′-TCAGCCTTGCGACCATACT-3′ and inserted into pMD18T to obtain pMD18T-18S. The fragments from non-conservative domain-encoding regions of 15 *CrCDPK* genes were amplified by PCR using *C. reinhardtii* complementary DNA (cDNA) as template. The fragments were digested with *Kpn*I/*Bam*HI and *Hin*dIII/*Sal*I and subsequently inserted into the corresponding cloning sites of pMD18T-18S to form vector pMD18T-CrCDPKF-18S-CrCDPKR, which contained an inverted repeat sequence of *CrCDPK* (CrCDPK IR). The vector pMD18T-CrCDPKF-18S-CrCDPKR was double digested with *Kpn*I and *Hin*dIII to obtain *CrCDPK* IR. Finally, the fragment of *CrCDPK* IR was inserted as a blunt-end fragment into *Eco*RI digested pMaa7 IR/XIR to give pMaa7 IR/*CrCDPK* IR.

The transformation of *C. reinhardtii* strain CC425 was performed by the glass bead procedure as described by Kindle (1990). The algal cells were cultured in TAP medium to a cell density of 2 × 10^6^ cells/ml, collected by centrifugation (3,000 *g*, 5 min), and resuspended in TAP medium to a cell density of 2 × 10^8^ cells/ml. Approximately 10 μg of plasmid DNA was added in a mixture containing 400 μl of algal cells and 300 mg of sterile glass beads. The reaction was mixed for 20 s on a bench-top vortex and allowed to recover for 2 days before plating at selective conditions. Maa7 IR/XIR and Maa7 IR/*CrCDPK* IR transformants were selected on TAP agar medium containing 1.5 mM of l-tryptophan, 5 μg/ml of paromomycin, and 5 μM of indole analog 5-fluoroindole (5-FI) as described by Rohr et al. (2004). Plates were incubated under dim light (approximately 50 μmol·m^−2^·s^−1^). Isolated transgenic strains were kept under constant selective pressure to prevent the loss of integrated IR transgenes. The diagram of RNAi vector Maa7 IR/XIR is shown in **Figure S1**. Because the IR corresponding to the target gene is internal to the arms of the Maa7 IR, efficient *Maa7* gene silencing may result in an efficiently suppressed target gene. Only the transgenic algal strains exhibiting *Maa7* and target gene silencing can grow on TAP medium with 5-FI and tryptophan because the *Maa7* gene encodes tryptophan synthase β-subunit, which can convert 5-FI into the toxic tryptophan 5-FI. Therefore, Maa7-silencing transgenic algal cells are resistant to 5-FI.

### Measurement of Oil Content, Cell Density, and Chlorophyll Content of RNAi Strains

For oil content measurement, each transformant was inoculated into HSM agar plates containing 0.5 mM of ammonium. After 12 days of cultivation, transgenic lines were transferred into a 96-well plate containing 200 μl of sterile water, and the oil content was measured as mentioned above. The cell density was determined by measuring the optical density of samples at 490 nm (OD490). To produce the standard curve, a series of *C. reinhardtii* samples of different cell densities were collected, and their OD490 values and cell densities were determined gravimetrically after plotting the standard curve of OD490 versus cell density (cell/ml). Samples were diluted by using appropriate ratios to ensure that the measured OD490 values were in the range of 0.15–0.75, if applicable. Cell density was calculated by using the following formula: cell density (cell/ml) = 2 × 10^7^ × OD490-669662 (*R*^2^ = 0.9996). *C. reinhardtii* transformed with the empty vector Maa7 IR/XIR (Maa7) was used as control. A total of 192 transgenic lines were selected to determine the oil content of each *CrCDPK* gene, and the boxplot was drawn by R3.5.1 software. Statistical analysis was performed using the SPSS software by means of one-way ANOVA and Duncan’s multiple range tests.

*CrCDPK3*-, *CrCDPK5*-, and *CrCDPK7*-RNAi-silencing algal transformants were selected for log-phase culture. *Maa7* empty vector transgenic lines were used as control. Ten transgenic lines were used for each *CrCDPK* gene. Maa7 and RNAi strains grown on a TAP agar plate were inoculated into 100-ml Erlenmeyer flasks containing 50 ml of HSM media with 0.5 mM of ammonium (N-limited). All cultures were placed in an incubator shaker maintained at 230 rpm and 24 °C and exposed to continuous illumination at a light intensity of 150 μmol·m^−2^·s^−1^. Samples for cell density, chlorophyll content, and oil content measurement were collected after 1, 2, 3, 4, and 5 days. Chlorophyll content was measured as described by Li et al. (2012b). Statistical analysis was performed using SPSS software by means of one-way ANOVA and Duncan’s multiple range tests.

## Results

### Genome-Wide Identification of *CDPK* Gene Family in *C. reinhardtii*

**Figure 1:**
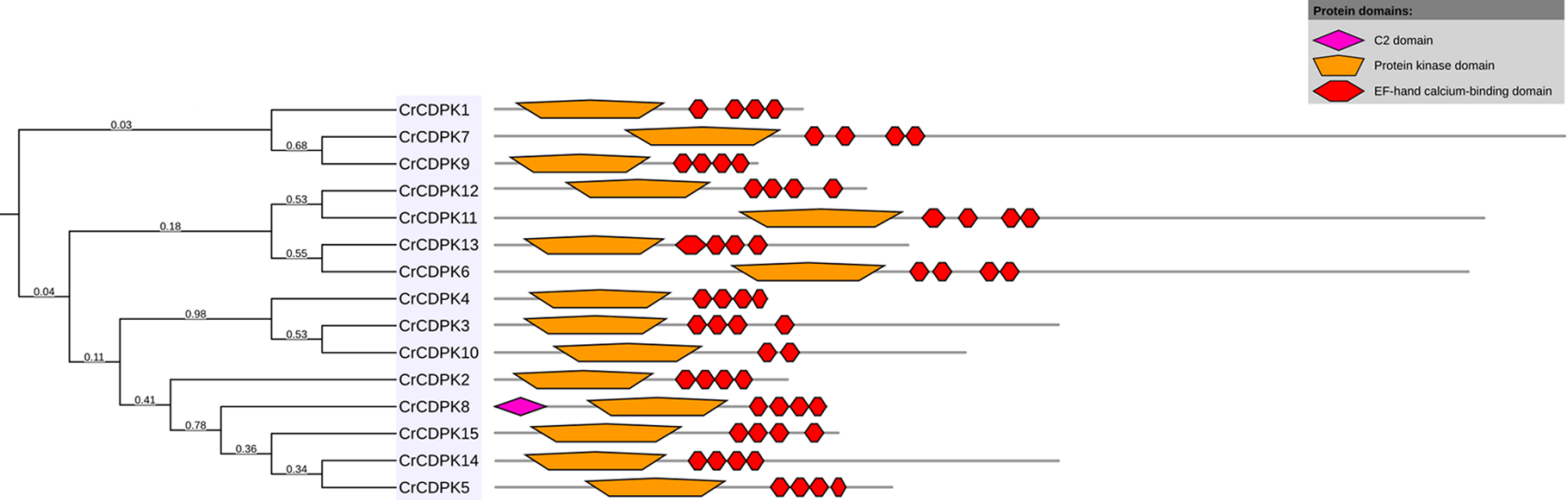
Conserved motif composition of *CrCDPK*s. The pink, orange, and red boxes indicate the C2, protein kinase, and elongation factor (EF)-hand calcium-binding domains.

BLAST searches of the *C. reinhardtii* genome were performed using *Arabidopsis* and rice *CDPK* gene sequences as queries. A total of 15 non-redundant *CrCDPK* genes were identified and designated as *CrCDPK1*–*CrCDPK15* (**Table 1**), all of which had domains typical for the CDPK family, which include a variable N-terminal domain, Ser/Thr kinase domain, autoinhibitory junction domain, and CaM-like domain (**Figure 1**). All *CrCDPK*s have four EF-hand motifs except for *CrCDPK10*, which lacked two EF-hands in the CaM-like domain (**Figure 1**). Eight *CrCDPK*s contain the predicted myristoylation sites at their N-terminus (**Table 1**). The open reading frame length of *CrCDPK*s ranged from 1,455 bp (*CrCDPK9*) to 5,940 bp (*CrCDPK7*), which encoded polypeptides in the range of 484 to 1,979 aa with predicted protein molecular masses from 54 to 198.9 kDa (**Table 1**). As shown in the table, the predicted isoelectric points of *CrCDPK*s ranged from 5.23 to 8.61. All of them tended to be acidic, except for *CrCDPK11* (8.61) and *CrCDPK6* (7.26).

**Table 1:** List of the 15 *CDPK* genes identified in *Chlamydomonas reinhardtii* and their sequence characteristics.

Gene name	Transcript name	Chromosome position	ORF	Amino acids	MW (kDa)	pI	EF-hand motifs (ScanProsite)	EF-hand motifs (SMART)	N-myristoylation site
CrCDPK1	Cre03.g144484.t1.1	3:267426…273277F	1,710	569	61.5	5.53	4	4	no
CrCDPK2	Cre19.g750597.t1.1	scaffold_19:78930…89155F	1,626	541	61.0	5.8	4	4	yes
CrCDPK3	Cre13.g564500.t1.1	13:392824…400949F	3,129	1,042	109.8	6.86	4	4	yes
CrCDPK4	Cre13.g571700.t1.1	13:1366004…1370421F	1,515	504	56.3	5.94	4	4	yes
CrCDPK5	Cre01.g003524.t1.1	1:653607…662145R	2,202	733	78.6	6.03	4	3	yes
CrCDPK6	Cre02.g074370.t1.2	2:163680…173615R	5,403	1,800	180.0	7.26	4	4	no
CrCDPK7	Cre02.g106650.t1.1	2:5287410…5296386F	5,940	1,979	198.9	6.98	4	4	yes
CrCDPK8	Cre17.g705000.t1.2	17:1209839…1214698F	1,842	613	67.4	5.54	4	4	no
CrCDPK9	Cre07.g328900.t1.2	7:2426734…2431805F	1,455	484	54.0	5.83	4	4	no
CrCDPK10	Cre12.g527000.t1.2	12:5121746…5126621F	2,613	870	92.1	6.78	2	2	no
CrCDPK11	Cre10.g418900.t1.2	10:161899…172025F	5,490	1,829	184.2	8.61	4	4	yes
CrCDPK12	Cre08.g382800.t1.2	8:4548983…4557157F	2,061	686	75.8	5.78	4	4	no
CrCDPK13	Cre01.g009500.t1.2	1:1763026…1771764R	2,295	764	82.6	6.41	4	4	yes
CrCDPK14	Cre02.g114750.t1.2	2:6297945…6306066F	3,126	1,041	107.3	5.82	4	4	yes
CrCDPK15	Cre06.g265550.t1.2	6:2117499…2123655F	1,908	635	68.0	5.23	4	3	no

ORF, open reading frame; EF, elongation factor.

### *CDPK* Gene Phylogenetic Analysis, Chromosomal Distribution, and Gene Structure in *C. reinhardtii*

**Figure 2:**
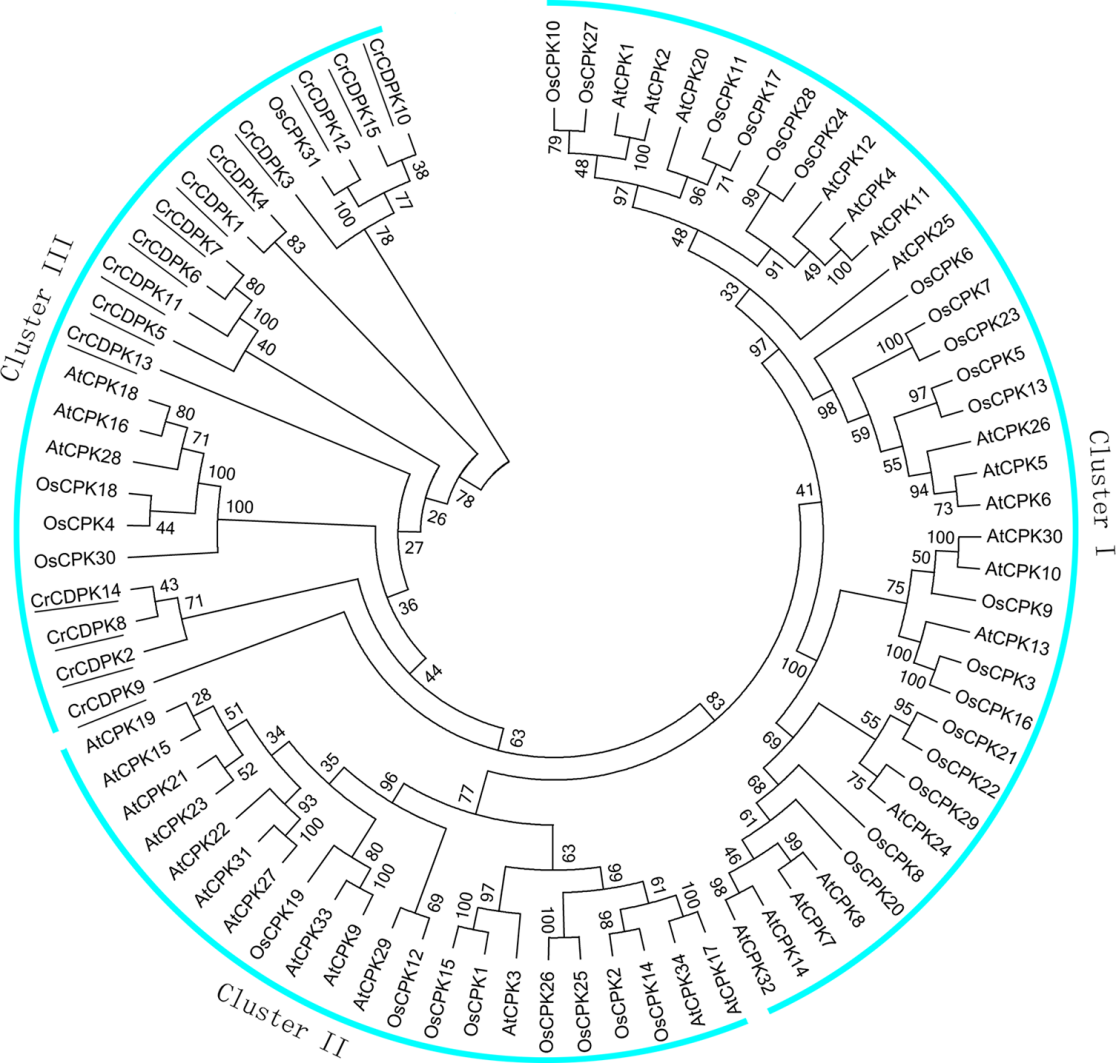
Phylogenetic tree of calcium-dependent protein kinase (CDPK) genes in *Chlamydomonas reinhardtii*, *Arabidopsis*, and rice. The phylogenetic tree was created according to the neighbor-joining method using Clustal X 2.0 and MEGA 6.0 software with 1,000 bootstraps on the basis of the amino acid sequence of 15 *CrCDPK*s (indicated with a black line), 34 AtCPKs, and 31 OsCPKs.

To evaluate the evolutionary relationships of CDPK family members among *C. reinhardtii*, *Arabidopsis*, and rice, 80 full-length amino acid sequences, including 15 *CrCDPK*s, 34 AtCPKs, and 31 OsCPKs, were used to generate a phylogenetic tree. The results showed that these CDPKs could be divided into three different clusters (I, II, and III) (**Figure 2**). Cluster I, the largest group, contained 18 AtCPKs and 19 OsCPKs. Cluster II included 13 AtCPKs and 8 OsCPKs. Interestingly, all CrCDPKs were assigned to Cluster III with a subclade of three AtCPKs and four OsCPKs. Additionally, *CrCDPK12* had high similarity with OsCPK31 (**Figure 2**). The *CrCDPK* gene family of Cluster III was clearly separated from the other two clusters with a strong bootstrap support, suggesting that this family probably came from a CDPK ancestor with *Arabidopsis* and rice but had a distant relationship with them.

The chromosomal localization of *CDPK*s indicated that 14 *CrCDPK*s were distributed among 10 chromosomes in *C. reinhardtii* and one *CrCDPK* was located on scaffold_19 (**Figure S2**). Seven chromosomes had one unique *CDPK* gene each. *CrCDPK1*, *CrCDPK15*, *CrCDPK9*, *CrCDPK12*, *CrCDPK11*, *CrCDPK10*, and *CrCDPK8* were located on chromosomes 3, 6, 7, 8, 10, 12, and 17, respectively. Chromosomes 1 and 13 had two *CDPK* genes each. Chromosome 1 carried *CrCDPK5* and *CrCDPK13*, whereas chromosome 13 carried *CrCDPK3* and *CrCDPK4*. *CrCDPK6*, *CrCDPK7*, and *CrCDPK14* were mapped on chromosome 2 (**Figure S2**). *CDPK* genes were widely distributed in the genome, implying a multiple-gene function of this family in *C. reinhardtii*. In addition, the *CrCDPK* gene cluster was not identified in the *C. reinhardtii* genome, suggesting that a few duplication events occurred in this gene family.

**Figure 3:**
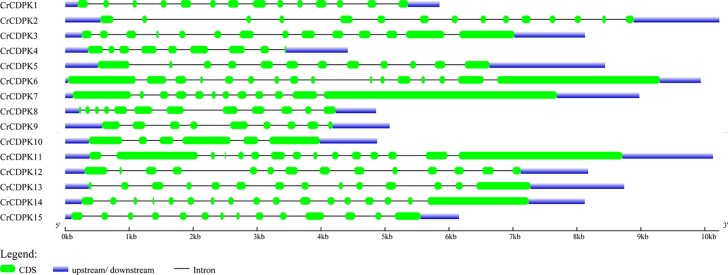
Gene structure of the *CDPK* gene family in *Chlamydomonas reinhardtii*. Blue and green bars represent untranslated regions (UTRs) and exons, respectively, and black strings represent introns.

The exon and intron structures of *CrCDPK* genes were visualized by using GSDS. As shown in **Figure 3**, all *CrCDPK*s contained 9–17 exons except for *CrCDPK10*, which had only six exons. *CrCDPK14* had a maximum of 17 exon regions. *CrCDPK4* and *CrCDPK9* both contained nine exons, and *CrCDPK5*, *CrCDPK7*, and *CrCDPK8* contained 12 exons. Fourteen exons were contained in five genes, namely, *CrCDPK1*, *CrCDPK3*, *CrCDPK12*, *CrCDPK13*, and *CrCDPK15*. *CrCDPK2* and *CrCDPK11* contained 15 exons, and *CrCDPK6* contained 16 exons.

### Expression Profiles of *CrCDPK*s in *C. reinhardtii* Against Various Stresses, Including N, P, and Fe Deficiencies and Cold and Salt Stresses

**Figure 4:**
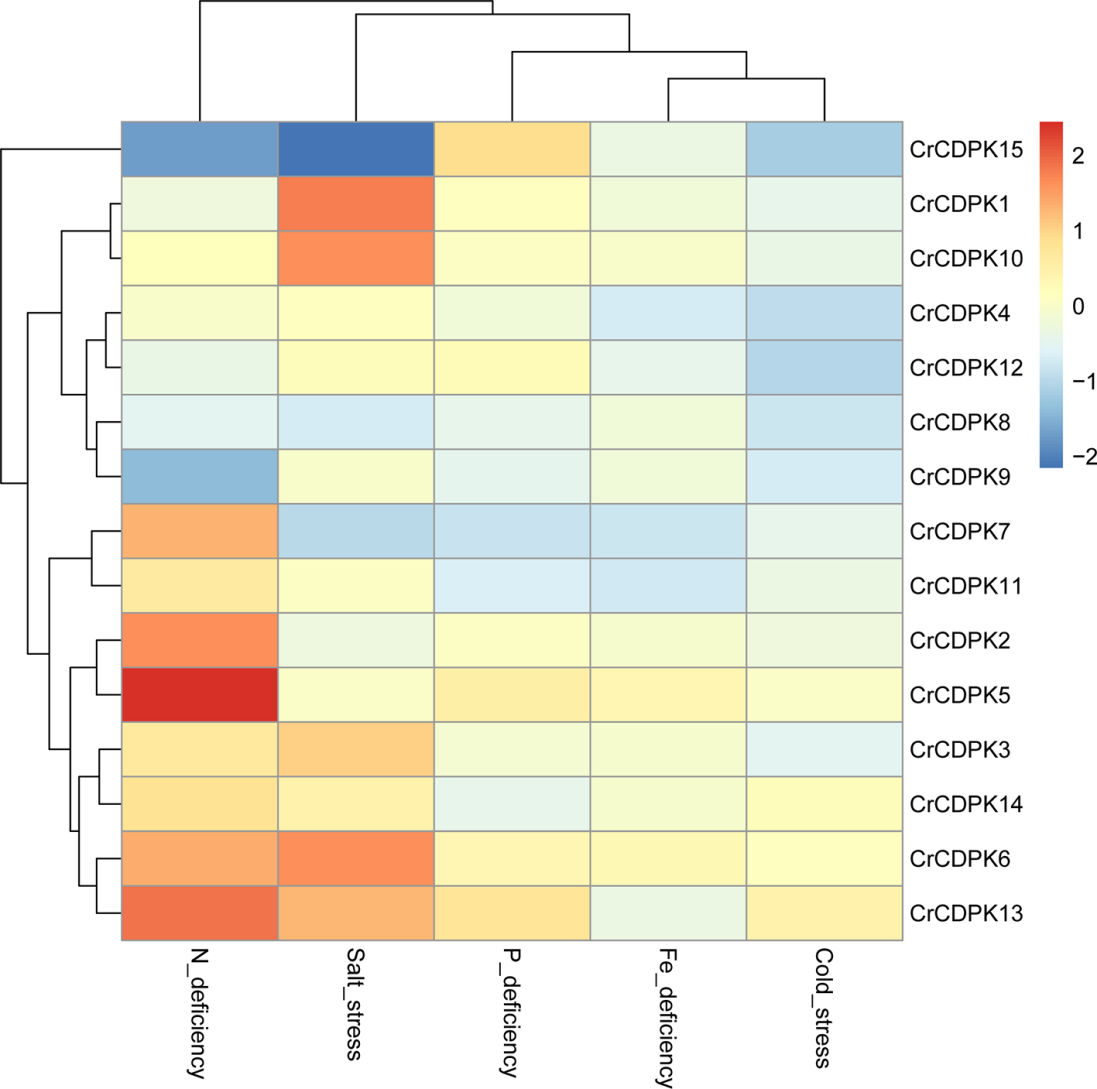
Expression profiles of 15 *CrCDPK* genes under nitrogen (N), phosphorus (P), and iron (Fe) deficiencies and cold and salt stresses. The algal cells were treated with various abiotic stresses and collected after 24 h for RNA-Seq. “Cold_stress” means that algal cells were exposed to 16 °C. The fold change value of each stress treatment compared with the control was normalized to complete hierarchical clustering analysis. Each *CrCDPK* gene was represented in a single row, and log_2_ (fold change) of each treatment was represented in a single column. Red and blue indicated higher and lower gene expression levels of *CrCDPK*s, respectively, in response to stress treatments compare with those of the control. The key is shown at the right side of the heat map.

To evaluate the expression profiles of the *CrCDPK* genes under stress conditions, algal cells treated with N, P, and Fe deficiencies and cold and salt stresses for 24 h were collected for RNA sequencing. We normalized the fold changes (stress treatment/control) of *CrCDPK*s and subjected them to hierarchical cluster analysis. As shown in **Figure 4**, except *CrCDPK15*, the expression profiles of *CDPK* genes can be divided into seven groups, wherein each group contained two genes. *CrCDPK1* and *CrCDPK10*, *CrCDPK4* and *CrCDPK12*, *CrCDPK8* and *CrCDPK9*, *CrCDPK7* and *CrCDPK11*, *CrCDPK2* and *CrCDPK5*, *CrCDPK3* and *CrCDPK14*, and *CrCDPK6* and *CrCDPK13* shared similar expression patterns under various stress conditions.

N deficiency and salt stress activated more differentially expressed *CrCDPK* genes than the other treatments (**Figure 4**). The log_2_ (fold change) values of *CrCDPK* genes under various stress conditions are shown in **Table 2**. The algal cells grown in normal conditions were treated as control. When FDR ≤0.05 and |log_2_ (fold change)| ≥1 were applied as filter conditions, five genes (*CrCDPK2*, *CrCDPK5*, *CrCDPK6*, *CrCDPK7*, and *CrCDPK13*) and two genes (*CrCDPK9* and *CrCDPK15*) were upregulated and downregulated, respectively, by N deficiency. In the case of salt stress, five genes (*CrCDPK1*, *CrCDPK3*, *CrCDPK6*, *CrCDPK10*, and *CrCDPK13*) were upregulated, and one gene (*CrCDPK15*) was downregulated. Moreover, *CrCDPK6* and *CrCDPK13* were upregulated, and *CrCDPK15* was downregulated by both N deficiency and salt stress treatment. By contrast, P and Fe deficiencies and cold stress caused a slight upregulation or downregulation of *CrCDPK* genes.

**Table 2:** The log_2_ (fold change) value of 15 *CrCDPK*s in various stress treatments compared to the control.

Gene_name	N_deficiency	Salt_stress	P_deficiency	Fe_deficiency	Cold_stress
CrCDPK1	−0.25	1.78	0.11	−0.18	−0.42
CrCDPK2	1.65	−0.28	0.09	−0.06	−0.23
CrCDPK3	0.70	1.02	−0.09	−0.08	−0.51
CrCDPK4	0.00	0.14	−0.21	−0.72	−0.94
CrCDPK5	2.45	0.04	0.53	0.37	0.05
CrCDPK6	1.36	1.65	0.34	0.31	0.10
CrCDPK7	1.33	−0.98	−0.83	−0.82	−0.41
CrCDPK8	−0.54	−0.72	−0.41	−0.22	−0.80
CrCDPK9	−1.37	−0.03	−0.49	−0.20	−0.71
CrCDPK10	0.19	1.63	0.06	−0.03	−0.38
CrCDPK11	0.62	0.10	−0.68	−0.76	−0.34
CrCDPK12	−0.39	0.19	0.27	−0.44	−1.03
CrCDPK13	1.89	1.26	0.79	−0.35	0.43
CrCDPK14	0.79	0.47	−0.41	−0.07	0.21
CrCDPK15	−1.73	−2.15	0.91	−0.32	−1.14

**Figure 5:**
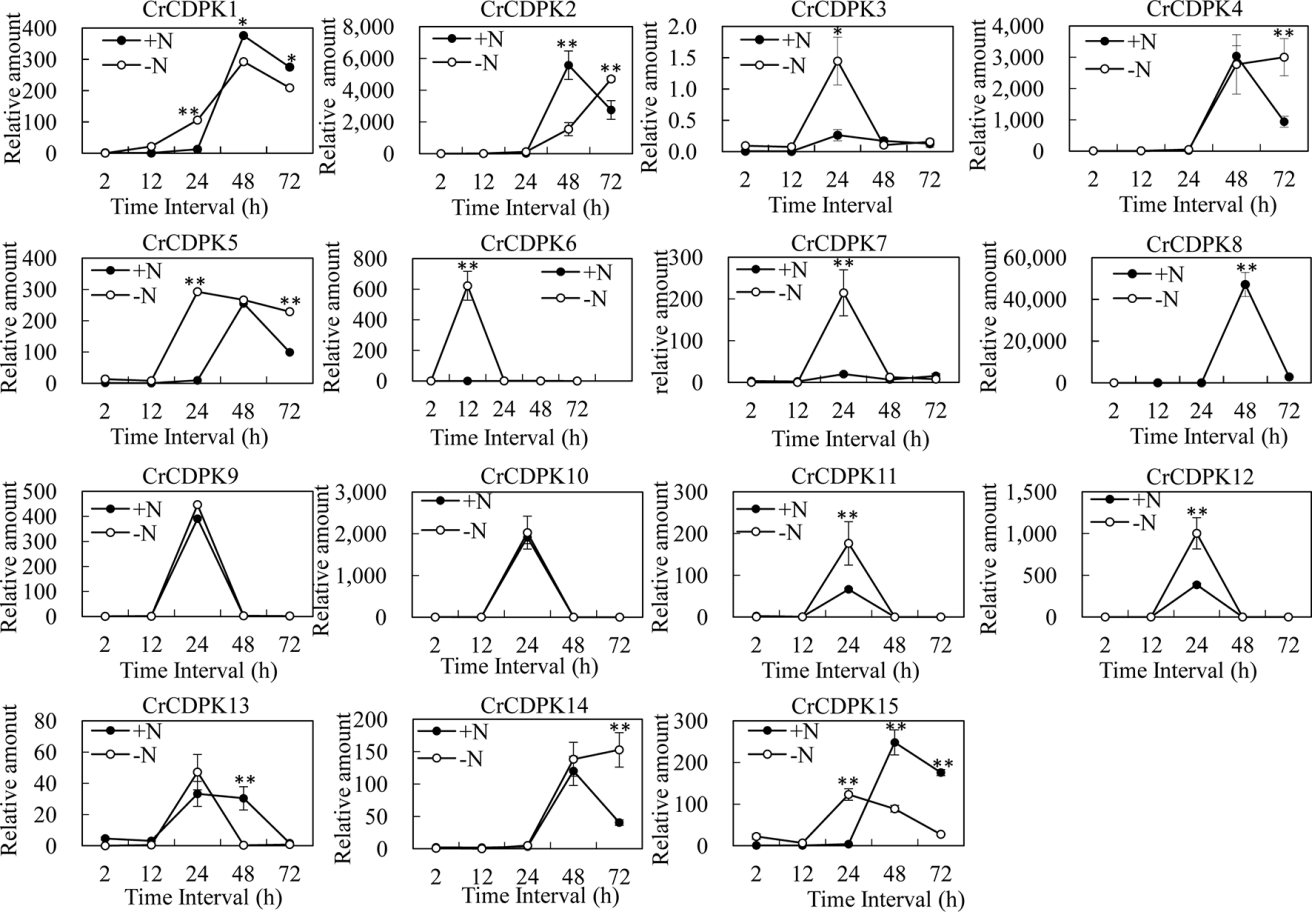
Expression profiles of 15 *CrCDPK* genes under normal and nitrogen-deficient conditions. Two-step cultivation was used for nitrogen deficiency treatment. The algal cells were first grown in Tris–acetate–phosphate (TAP) medium for 3 days to reach a growth plateau and collected by centrifugation (3,000 *g*). Finally, the cultured cells were divided into two groups: one group was inoculated into high salt minimal (HSM) medium without nitrogen (HSM-N), and the other was inoculated into normal HSM that was used as control. The samples for quantitative reverse transcription (qRT)-PCR were collected after 0, 2, 12, 24, 48, and 72 h. All expression level values were normalized to the value of the 18S gene level. The data shown are means ( ± SD, *n* = 3). Significance is indicated as *p < 0.05 and **p < 0.01 (*t*-test). This experiment was repeated three times with similar results.

To learn more about the expression profiles of *CrCDPK* genes under N-deficient conditions, the algal cells were sampled after 2, 12, 24, 48, and 72 h of N-deficient treatment, and the untreated cells were used as control. The expression patterns of *CrCDPK*s in *C. reinhardtii* were examined through qRT-PCR (**Figure 5**). Compared with the control, all *CrCDPK* genes were found to be not significantly differentially expressed after 2 h. The transcript level of *CrCDPK6* was remarkably increased after 12 h and reached its peak, which was approximately 600 times higher than that of control (**Figure 5**). The expression levels of seven *CrCDPK* genes were significantly induced by N deficiency after 24 h. Among them, four *CrCDPK*s (*CrCDPK3*, *CrCDPK7*, *CrCDPK11*, and *CrCDPK12*) showed similar expression patterns, which peaked after 24 h. *CrCDPK1* and *CrCDPK15* were dramatically upregulated after 24 h of N deficiency but significantly downregulated after 48 and 72 h. *CrCDPK5* was remarkably activated after 24 h and continually expressed up to 72 h (**Figure 5**). Interestingly, three genes (*CrCDPK2*, *CrCDPK8*, and *CrCDPK13*) showed lower expression levels than the control after 48 h, and two genes (*CrCDPK4* and *CrCDPK14*) were significantly upregulated after 72 h (**Figure 5**). The expression profile of the *CrCDPK* gene family suggested that most *CrCDPK*s showed earlier expression and/or higher transcription abundance under N-deficient conditions.

### Effect of LaCl_3_ on N and P Deficiency-Induced Oil Accumulation in *C. reinhardtii*

**Figure 6:**
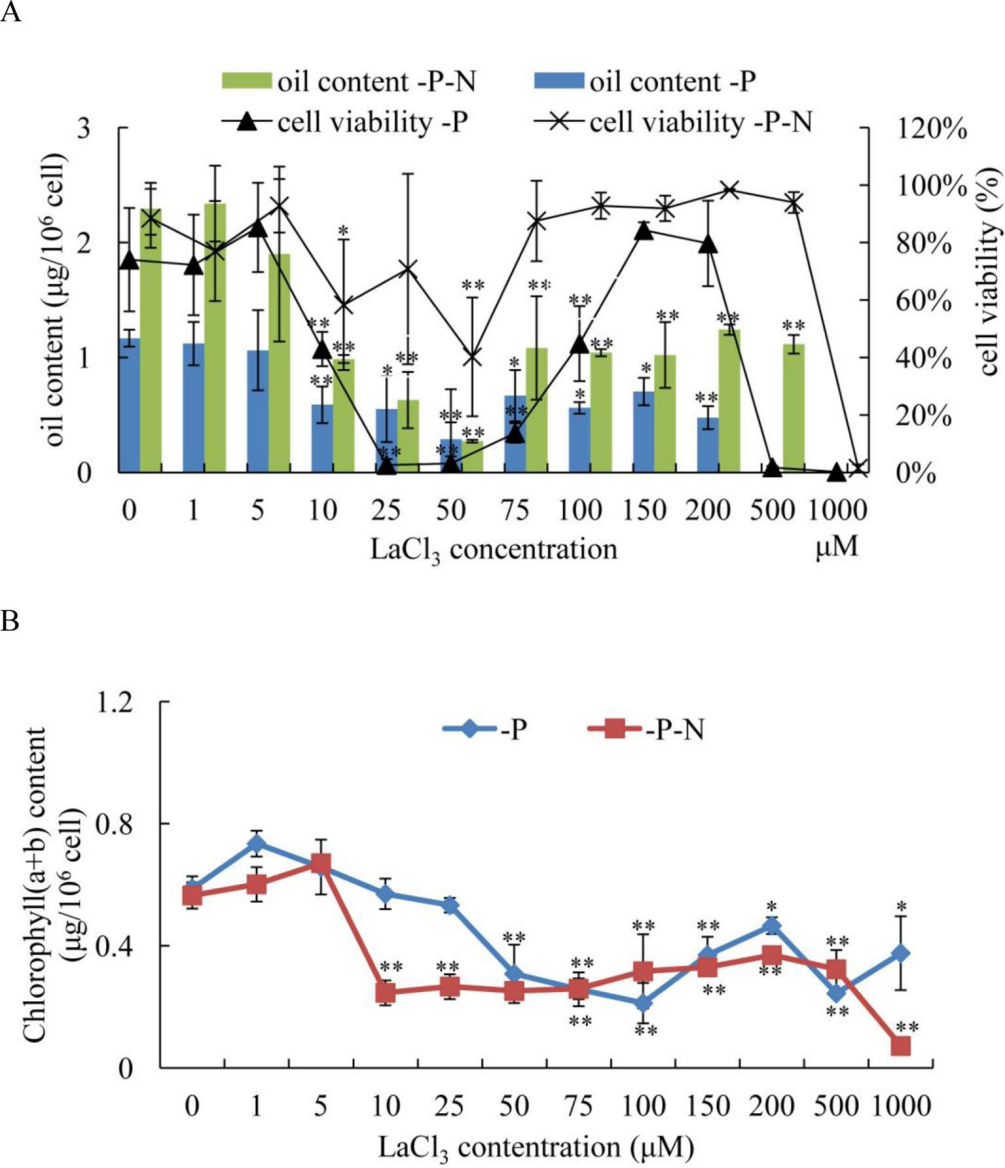
Effects of lanthanum chloride (LaCl_3_) on oil accumulation **(A)** and chlorophyll content **(B)** of *Chlamydomonas reinhardtii* cells for 48 h under P- and N-and-P-deficient conditions. **(A)** Algal cells were grown in high salt minimal (HSM) media for 3 days to reach a growth plateau and collected by centrifugation. Finally, the cultured cells were inoculated into P- and N-and-P-deficient HSM media but with various concentrations of LaCl_3_ (0, 1, 5, 10, 25, 50, 75, 100, 150, 200, 500, and 1,000 µM). The oil contents and cell viability of the samples were measured at 48 h after treatment with different concentrations of LaCl_3_. Fluorescein diacetate [FDA; 0.01% (w/v)]-stained cells were counted by flow cytometry using CyFlow Cube 6. Cellular viability was presented as percentages of FDA-stained live cells to total 10^4^ cell numbers. “0 µM” means LaCl_3_-untreated cells (control). Data shown are means ( ± SD, *n* = 3). Asterisks indicate statistically significant differences compared with the corresponding control (Duncan’s multiple range test: *p < 0.05, **p < 0.01). This experiment was repeated three times with similar results.

To determine whether the Ca^2+^ signaling pathway played an important role in N deficiency-induced oil accumulation in *C. reinhardtii*, LaCl_3_, a general plasma membrane Ca^2+^ blocker, was used to prevent the Ca^2+^ influx in N deficiency-treated algal cells. Because phosphate and La^3+^ formed precipitates, the medium for N deficiency treatment (HSM-N) did not contain P. Algal cells were grown in TAP media for 3 days, collected by centrifugation, and inoculated into HSM-P and HSM-N-P media with various concentrations of LaCl_3_ (1, 5, 10, 25, 50, 75, 150, 100, 200, 500, and 1,000 μM). At different treatment times, cellular viability, chlorophyll content, and oil content of the samples were measured. Algal cells treated with P or N and P deficiency and 0 μM of LaCl_3_ were used as control. The results showed that compared with those in untreated algal cells, the oil contents in *C. reinhardtii* cells dramatically decreased after treating with over 10 μM of LaCl_3_ for 48 h under P or N and P starvation (**Figure 6A**). Algal cells treated with 10–50 μM of LaCl_3_ displayed lower oil content than those treated with other concentrations, suggesting that 10–50 μM LaCl_3_ is toxic to algal cells (**Figure 6A**). Because LaCl_3_ toxicity is not dosage dependent (**Figure S3**), the cell viabilities did not significantly change under 75–500 μM of LaCl_3_ and N and P starvation. Although the oil content significantly decreased, results suggested that Ca^2+^ contributed to N and P deficiency-induced oil accumulation in *C. reinhardtii*. LaCl_3_ had a significant effect on the chlorophyll content of *C. reinhardtii* (**Figure 6B**). After the application of over 50 μM of LaCl_3_, chlorophyll (a + b) contents were significantly decreased under P-deficient conditions. By contrast, treatment with over 10 μM of LaCl_3_ induced a dramatic decline in the chlorophyll content of algal cells grown in N-and-P-deficient media.

### RNAi Silencing of *CrCDPK* Genes Altered the Oil Contents of N-Starved Cells in *C. reinhardtii*

**Figure 7:**
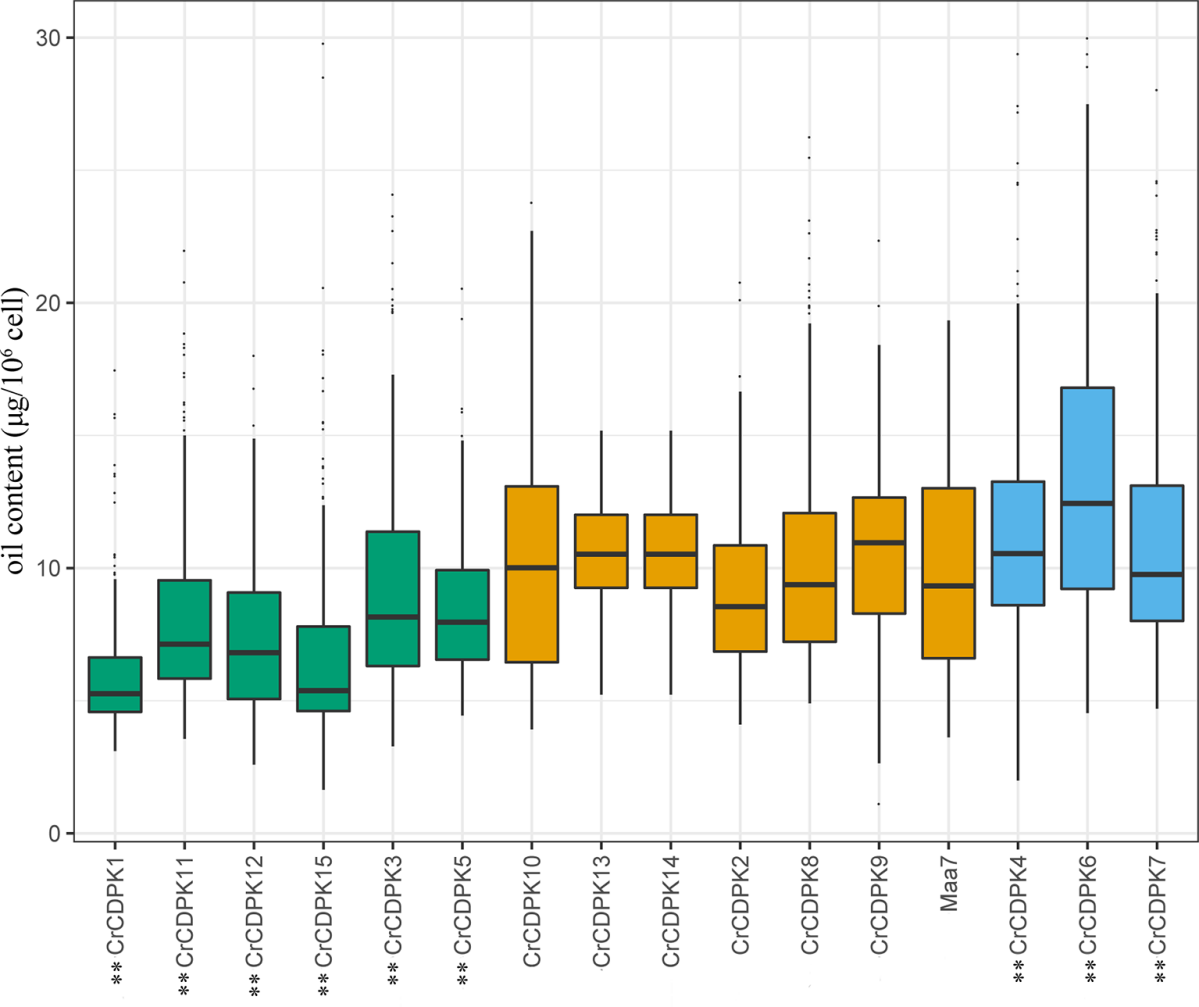
The oil contents of *CrCDPK*-silenced cells of *Chlamydomonas reinhardtii* grown under N-deficient conditions. *CrCDPK* RNA interference (RNAi)-silencing transgenic lines were inoculated into Tris–acetate–phosphate (TAP) agar medium containing 0.5 mM of ammonium and transferred into a 96-well plate containing sterile water for the measurement of oil content after 12 days of cultivation. Boxplots display medians with first and third quartiles, whereas whiskers indicate the smallest and largest non-outlier observations (*n* = 192). Maa7 is *Chlamydomonas reinhardtii* transformed with the empty vector Maa7/XIR, which was used as control. An independent-samples *t*-test was performed for statistical analysis. Significance is indicated as **p < 0.01.

To understand the function of *CrCDPK* genes in N deficiency-induced oil accumulation in *C. reinhardtii*, RNAi-mediated gene silencing was performed for all 15 *CrCDPK*s. A total of 192 transgenic lines were generated in each case, and their oil contents were determined under N-deficient conditions. Transgenic cells containing RNAi plasmid Maa7 IR/XIR were used as control. The results are shown in **Figure 7**. Compared with those of the control, the oil contents of algal cells transformed with *CrCDPK1*, *CrCDPK3*, *CrCDPK5*, *CrCDPK11*, *CrCDPK12*, and *CrCDPK15* significantly decreased after N deficiency treatment, indicating that these genes play positive roles in N deficiency-induced oil accumulation in *C. reinhardtii*. By contrast, transgenic lines carrying the small interfering RNA (siRNA) against *CrCDPK4*, *CrCDPK6*, and *CrCDPK7* exhibited a dramatic increase in oil content (**Figure 7**), implying that these three genes are negatively involved in oil accumulation under N-deficient conditions in *C. reinhardtii*. No significant changes were found in the cell densities between the RNAi strains and control (**Figure S4**). Additionally, no obvious difference was observed between the control and *CrCDPK2*-, *CrCDPK8*-, *CrCDPK9*-, *CrCDPK10*-, *CrCDPK13*-, and *CrCDPK14*-RNAi-silencing transgenic lines (**Figure 7**), suggesting that these genes are not involved in oil accumulation or that other members of *CrCDPK* act redundantly.

**Figure 8:**
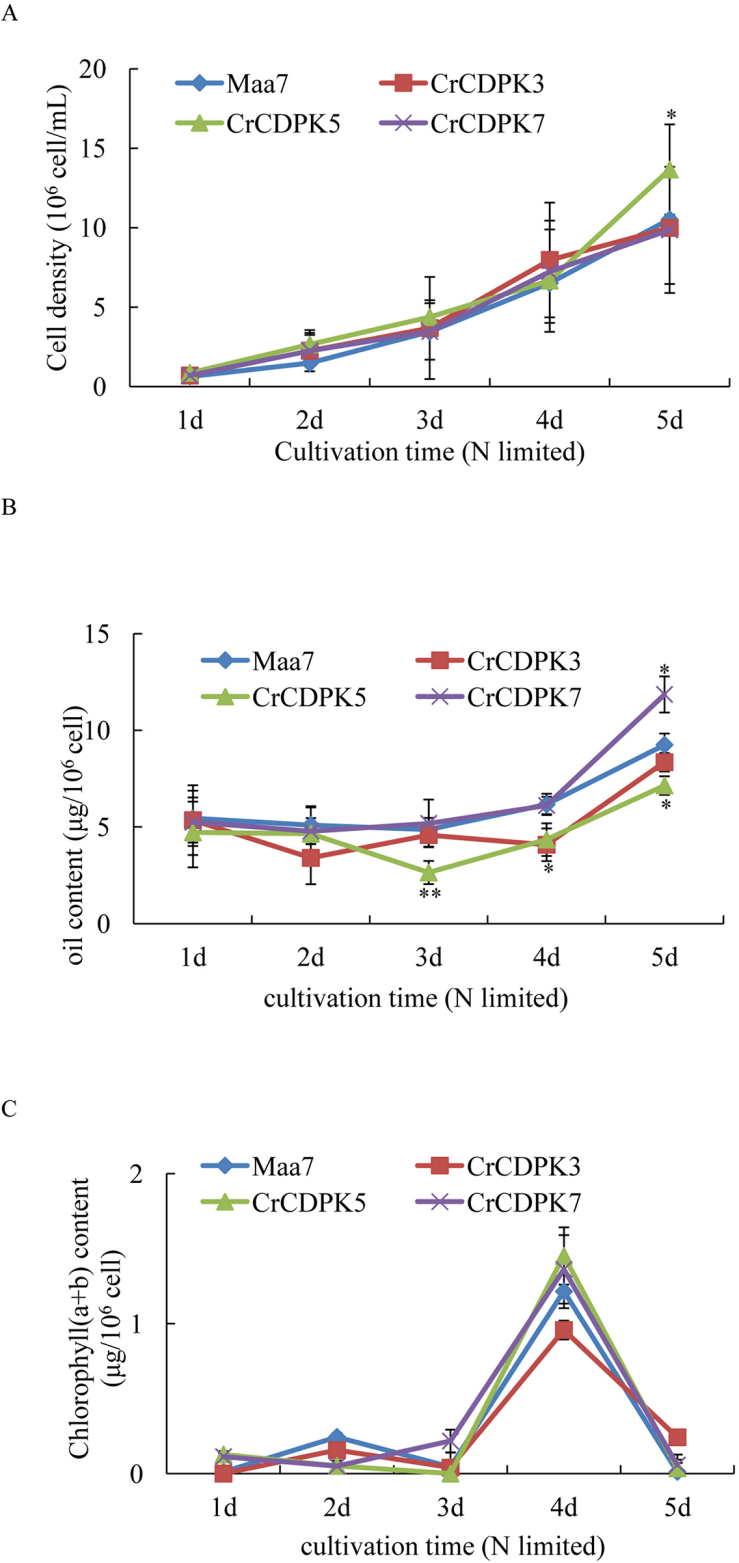
Cell density **(A)**, oil content **(B)**, and chlorophyll content **(C)** of *CrCDPK3*-, *CrCDPK5*-, and *CrCDPK7*-RNA interference (RNAi) strains. Maa7- and *CrCDPK*-RNAi strains grown on a Tris–acetate–phosphate (TAP) agar plate were inoculated into 100-ml Erlenmeyer flasks containing 50 ml of high salt minimal (HSM) media with 0.5 mM of ammonium (N-limited). Samples for cell density, chlorophyll content, and oil content measurement were collected after 1, 2, 3, 4, and 5 days. Statistical analysis was performed using the SPSS software by means of one-way ANOVA and Duncan’s multiple range tests. Data shown are means ( ± SD, *n* = 10). Asterisks indicate statistically significant differences compared with the corresponding control (Duncan’s multiple range test: *p < 0.05, **p < 0.01). This experiment was repeated three times with similar results.

To validate the oil changes in RNAi strains, *CrCDPK3*-, *CrCDPK5*-, and *CrCDPK7*-RNAi-silencing algal transformants were selected for log-phase culture. *Maa7* empty vector transgenic lines were used as control. The results are shown in **Figure 8**. Under N-limited conditions, the cell density gradually increased from 1 to 5 days (**Figure 8A**), and the chlorophyll content sharply increased after 4 days and then decreased rapidly after 5 days (**Figure 8C**). No significant changes were observed in the cell density and chlorophyll content between the RNAi strains and control except for *CrCDPK5-*RNAi cells, which showed more cell density than the control. On the contrary, compared with the control, *CrCDPK5*-RNAi cells showed a significant decline in oil content from 3 to 5 days of N-limited cultivation, and *CrCDPK3*-RNAi cells exhibited lower oil content after 4 days. On the other hand, the oil content of *CrCDPK7*-RNAi was significantly increased after 5 days of cultivation (**Figure 8B**). This result was consistent with those obtained above.

## Discussion

Microalgae tend to accumulate oils in response to different environmental stresses with a slow growth rate. Several genetic strategies have been employed to obtain strains with high oil content and a rapid growth rate. However, they are inefficient due to the limited knowledge about the molecular mechanisms of stress-induced oil biosynthesis in microalgae. On the other hand, Ca^2+^ signaling is a core regulator of cellular responses to miscellaneous biotic and abiotic stresses (Dodd et al., 2010) and plays an important role in oil biosynthesis in mammals. Ca^2+^ imbalance may result in dysfunctions of adipose tissue in mammals. For example, a rise in cytosolic Ca^2+^ can promote TAG accumulation and storage in mice (Arruda and Hotamisligil, 2015). Lipid droplets may also accumulate in the liver, heart, and skeletal muscles of mice deficient in store-operated Ca^2+^ entry, which is a ubiquitous Ca^2+^ influx pathway (Maus et al., 2017). However, little information is available on the Ca^2+^ signaling pathway involved in stress-induced oil biosynthesis in microalgae. In the present study, LaCl_3_ at concentrations of 75 μM or higher has important consequences for N and P deficiency-induced oil accumulation in *C. reinhardtii*. After the application of LaCl_3_, the oil content of algal cells was significantly decreased compared to that of the control (**Figure 6**). As a plasma membrane calcium channel blocker, LaCl_3_ prevented Ca^2+^ influx *via* inhibiting Ca^2+^-ATPase activity. The results indicated that changes in Ca^2+^ homeostasis directly affected stress-induced oil accumulation in *C. reinhardtii*, in which Ca^2+^ is an important contributing factor.

Several Ca^2+^ sensors, including CaM and CaM-like proteins, CBL, and CDPKs, have been identified in organisms. However, only CDPKs display both Ca^2+^ sensing and protein kinase domains within a single protein. Thus, they can directly translate Ca^2+^ signals into downstream protein phosphorylation without protein partners (Boudsocq and Sheen, 2013). In recent years, CDPKs have attracted increasing attention due to their special feature. In this study, we investigated the *CDPK* gene family in the *C. reinhardtii* genome by using *Arabidopsis* and rice CDPK sequences as queries. A total of 15 non-redundant *CrCDPK* genes were identified from *C. reinhardtii* (**Table 1**), which was consistent with the results obtained by Hamel et al. (2014). All *CrCDPK* genes harbor a typical structure containing protein kinase and Ca^2+^ binding domains in *C. reinhardtii* (**Figure 1**), and all of them contain four EF-hand motifs except for *CrCDPK10*, which only has two EF-hands. In other plant species, such as tomato and potato, similar results were obtained: some CDPK genes have less than four EF-hands (Gromadka et al., 2018; Hu et al., 2016a; Hu et al., 2016b). Boudsocq et al. (2012) reported that the *AtCPK25* gene is Ca^2+^ independent owing to the absence of functional EF-hands in *Arabidopsis*. The qRT-PCR results also showed that the expression level of *CrCDPK10* under N-deficient conditions was quite similar with that under normal conditions (**Figure 5**), implying that *CrCDPK10* probably has weak Ca^2+^-binding affinities and is insensitive to Ca^2+^ in *C. reinhardtii*.

In addition, eight *CrCDPK* genes were predicted to have myristoylation sites that play an essential role in membrane targeting and protein–protein interactions. On the basis of their phylogenetic relationships, all *CrCDPK*s were clustered into a distinct group along with a subclade of AtCPK16/AtCPK18/AtCPK28 and OsCPK4/OsCPK18/OsCPK30, whereas the remaining AtCPKs and OsCPKs were split into two other groups (**Figure 2**). The *AtCPK28* gene responds to osmotic stress and controls stem elongation and vascular development in *Arabidopsis* (Matschi et al., 2013; Gao et al., 2014). *OsCPK4* functions as a positive modulator of various abiotic stress and negatively regulates innate immunity in rice (Wang et al., 2018a). Because these known-function genes are involved in various physiological processes, the functions of *CrCDPK* genes are difficult to predict and remain unclear in *C. reinhardtii*.

To investigate the expression patterns of *CrCDPK* genes in response to abiotic stress, we determined their transcript levels in *C. reinhardtii* against N, P, and Fe deficiencies and salt and cold stresses. Interestingly, the expression profiles of *CrCDPK*s were paired except for *CrCDPK15* and varied greatly between treatments. About half of *CrCDPK*s were significantly induced by N deficiency or salt stress. By contrast, *CrCDPK* genes were insensitive to P and Fe deficiencies and cold stress because no significant changes were observed in their messenger RNA (mRNA) levels under such conditions (**Figure 4**). Many salt stress-responsive *CDPK* genes have been identified in higher plants in previous studies. For instance, *OsCPK12* positively regulates salt tolerance by reducing the accumulation of reactive oxygen species (Asano et al., 2012). Recently, *CDPK*s have been demonstrated to be related to N deficiency. The transcription levels of three *OtCDPK*s were upregulated by N depletion in marine green algae *Ostreococcus tauri* (Caló et al., 2017), and the mRNA levels of *CDPK1* and *CDPK3* from *C. reinhardtii* showed over 10-fold increase after 1 h of N starvation, followed by a decrease from 3 to 18 h (Motiwalla et al., 2014). These two *C. reinhardtii* CDPKs corresponded to *CrCDPK8* and *CrCDPK9* in our study. With the combination of the results of RNA sequencing and qRT-PCR analysis, *CrCDPK8* was significantly downregulated by N deficiency after 48 h (**Figure 5**), and *CrCDPK9* was significantly downregulated after 24 h (**Figure 4**).

As mentioned above, oil droplet biogenesis was prevented by over 75 μM of LaCl_3_ in *C. reinhardtii* (**Figure 6**), revealing that Ca^2+^ has an important regulatory role in N deficiency-induced oil accumulation. Ma et al. (2011) reported that TAG is dose-dependently decreased with increasing dietary calcium levels, and TAG transport protein is downregulated in hamster. Thus, the relationship between calcium and oil biosynthesis is still unclear. In higher plants, several CDPKs have been found to be associated with oil bodies in some oilseeds, such as sandalwood, cotton, and soybean, indicating that CDPK and Ca^2+^ may have a regulatory role during oil accumulation or oil body biogenesis (Anil et al., 2003). In the present study, to understand the functions of *CrCDPK*s in oil biosynthesis in *C. reinhardtii*, RNAi gene silencing was performed for all 15 *CrCDPK* genes. The transgenic cells containing the empty vector Maa7 XR/XIR were used as control. Six *CrCDPK*-silencing transgenic lines, especially *CrCDPK1*, exhibited lower levels of oil content than the control under N-deficient conditions (**Figure 7**). Oil content was significantly decreased by 39.02% after the *CrCDPK1* gene was silenced in *C. reinhardtii*. Notably, the results of qRT-PCR showed that all of these six *CrCDPK* genes were upregulated after 24 h in response to N deficiency (**Figure 5**). The data indicated that these six *CrCDPK* genes, which include *CrCDPK1*, *CrCDPK3*, *CrCDPK5*, *CrCDPK11*, *CrCDPK12*, and *CrCDPK15*, play positive roles in oil accumulation in *C. reinhardtii* under N-deficient conditions. The other three *CrCDPK*s (*CrCDPK4*, *CrCDPK6*, and *CrCDPK7*) showed higher levels of oil content than the control (**Figure 7**), suggesting that they play negative roles in oil accumulation. In addition, according to their expression patterns examined by qRT-PCR (**Figure 5**), they may be involved in the different stages of oil biosynthesis in N-deficient cells in *C. reinhardtii* because *CrCDPK4* was upregulated after 72 h, *CrCDPK6* after 12 h, and *CrCDPK7* after 24 h. The oil contents of the remaining six *CrCDPK*-silencing transgenic cells showed no significant changes compared with those of the control. This result was consistent with the observation that they were all insensitive to N deficiency at transcript levels (**Figure 5**).

In conclusion, there were at least 15 members of the *CDPK* gene family in the *C. reinhardtii* genome, and they all harbored the typical conserved domains, namely, the EF-hand Ca^2+^-binding domain and protein kinase domain. Phylogenetic analysis showed that these *CrCDPK*s were clustered into the same group, separating from most *Arabidopsis* and rice *CDPK* genes. The *CrCDPK* genes were widely distributed in the genome, and all of them contained 6–17 exons. Most *CrCDPK*s were transcriptionally insensitive to P and Fe deficiencies and cold stress after 24 h treatment, but half of them were significantly upregulated/downregulated by N deficiency and salt stress. Under N-deficient conditions, oil droplets disappeared in *C. reinhardtii* cells after application of over 100 μM of LaCl_3_. When the 15 *CrCDPK* genes were silenced in *C. reinhardtii*, six genes displayed lower levels of oil content, and three genes showed higher levels than the control. Correspondingly, nine *CrCDPK* genes were significantly upregulated by N deficiency at different times.

## Data Availability

The data generated in this study can be found in the NCBI using accession numbers SRX7003584, SRX7003583, SRX7003582, SRX7003581, SRX7003580.

## Author Contributions

YL and XD designed the experiments, analyzed the data, and wrote the manuscript. YL, XF, HD, JL, and WZ conducted the experiments.

## Funding

This work was financially supported by the National Natural Science Foundation of China (Grant No. 31770272) and Central Public-interest Scientific Institution Basal Research Fund for Chinese Academy of Tropical Agricultural Sciences (No. 1630052018001, No. 1630052016009, and No. 19CXTD-32).

## Conflict of Interest Statement

The authors declare that the research was conducted in the absence of any commercial or financial relationships that could be construed as a potential conflict of interest.
