# Supplementary material for: Genome-Wide Identification of Calcium-Dependent Protein Kinases in Chlamydomonas reinhardtii and Functional Analyses in Nitrogen Deficiency-Induced Oil Accumulation
Source: Front Plant Sci. 2019 Oct 22;10:1147. doi: 10.3389/fpls.2019.01147 (PMC6818280; doi:10.3389/fpls.2019.01147)
Supplement: Supplementary file 2 [file Table_2.docx]

| Table S2 The list of CrCDPK gene specific primers used for qRT-PCR. | |
| --- | --- |
|  |  |
| gene name | primer sequence |
| 18S gene | Forward primer: 5'-TCAACTTTCGATGGTAGGATAGTG-3';  Reverse primer: 5'-CCGTGTCAGGATTGGGTAATTT-3' |
| CrCDPK1 | Forward primer: 5'-CACCGCCGGACTGTACAACC-3';  Reverse primer: 5'-CCGTCGCCCAGGTATGCCAC-3' |
| CrCDPK2 | Forward primer: 5'-CCTGTCCGCCAAGCCCTG-3';  Reverse primer: 5'-AGGTCCATACTTCGCAGCAG-3' |
| CrCDPK3 | Forward primer: 5'-CGCCATCACACGCAAGG-3';  Reverse primer: 5'-GCCTGGACCATCATCTTGAGGAA-3' |
| CrCDPK4 | Forward primer: 5'-CCACCGCCGCCATCACAC-3';  Reverse primer: 5'-CCAGCACCGCCTCAATCTCG-3' |
| CrCDPK5 | Forward primer: 5'-GAAGGGCACCAACATCC-3';  Reverse primer: 5'-TGCTGCAAAAGCTCGTT-3' |
| CrCDPK6 | Forward primer: 5'-CCATGCGCGGCTCCAC-3';  Reverse primer: 5'-CGCCTCTTCCAGCTCCTTG-3' |
| CrCDPK7 | Forward primer: 5'-AACAACGGCGAGTGGGA-3';  Reverse primer: 5'-GCACCACGTCATCGAAC-3' |
| CrCDPK8 | Forward primer: 5'-GGCCTCATCGACTACAACG-3';  Reverse primer: 5'-AGTGCCTCGTTGTTGTTC-3' |
| CrCDPK9 | Forward primer: 5'-TGGACAGCGACCACAAC-3';  Reverse primer: 5'-CATCAGCTTGGCCTCCG-3' |
| CrCDPK10 | Forward primer: 5'-CCCACCGCCGTCATCG-3';  Reverse primer: 5'-CATGGTCAGGAACTCAGCGT-3' |
| CrCDPK11 | Forward primer: 5'-TCCCACCCGGAGTTGC-3';  Reverse primer: 5'-TACCGTCCTTGTCCATCTCG-3' |
| CrCDPK12 | Forward primer; 5'-AGATCAACCGCATGTTCGAG-3';  Reverse primer: 5'-TTGGCGAACTCCTGCT-3' |
| CrCDPK13 | Forward primer: 5'-CAGCAACGACCTGCACAAG-3';  Reverse primer: 5'-CGTCGTTGTTCTTGTCCAC-3' |
| CrCDPK14 | Forward primer: 5'-GTGGAGGAGTTCAGCGA-3';  Reverse primer: 5'-CATCATTGCCACGAACTC-3' |
| CrCDPK15 | Forward primer: 5'-GCTCGCTGCCGCACAT-3';  Reverse primer: 5'-AGCACTGACTTGACGACCAC-3' |
