# Supplementary material for: Genome-Wide Identification of Calcium-Dependent Protein Kinases in Chlamydomonas reinhardtii and Functional Analyses in Nitrogen Deficiency-Induced Oil Accumulation
Source: Front Plant Sci. 2019 Oct 22;10:1147. doi: 10.3389/fpls.2019.01147 (PMC6818280; doi:10.3389/fpls.2019.01147)
Supplement: Supplementary file 3 [file Table_3.docx]

Supplementary material ----Figure S


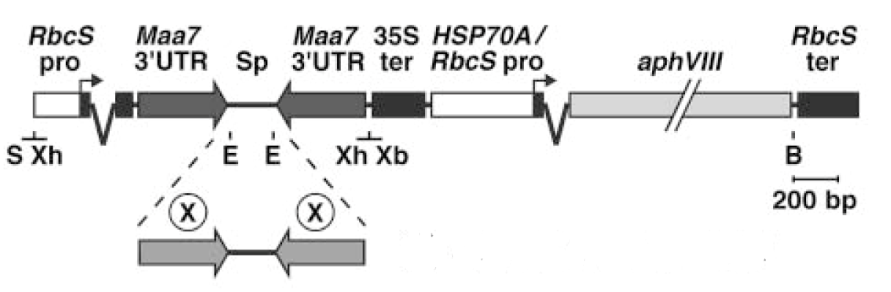


Figure S1 Diagram of the Maa7 IR/XIR RNAi construct.

X means target gene; Sp means a DNA spacer.

The fragments corresponding to target gene X was cloned in sense and antisense orientation, flanking a DNA spacer (Sp), in between the arms of the *Maa7* 3’UTR IR ( Rohr et al. (2004)).


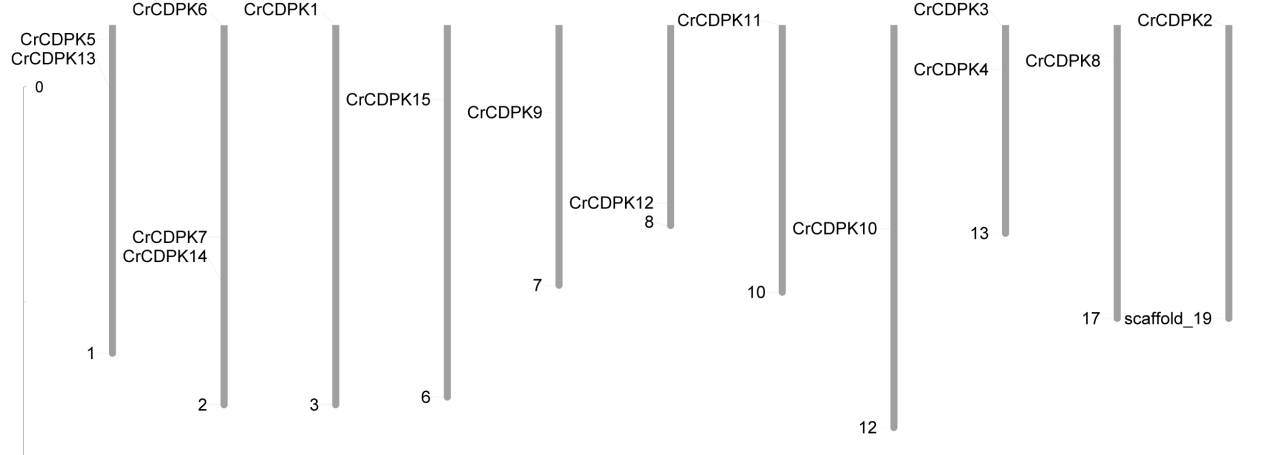


Fig. S2 Chromosomal distributions of CDPK genes in C.reinhardtii genome. The numbers at the bottom of each chromosome represent the number of the chromosomes.


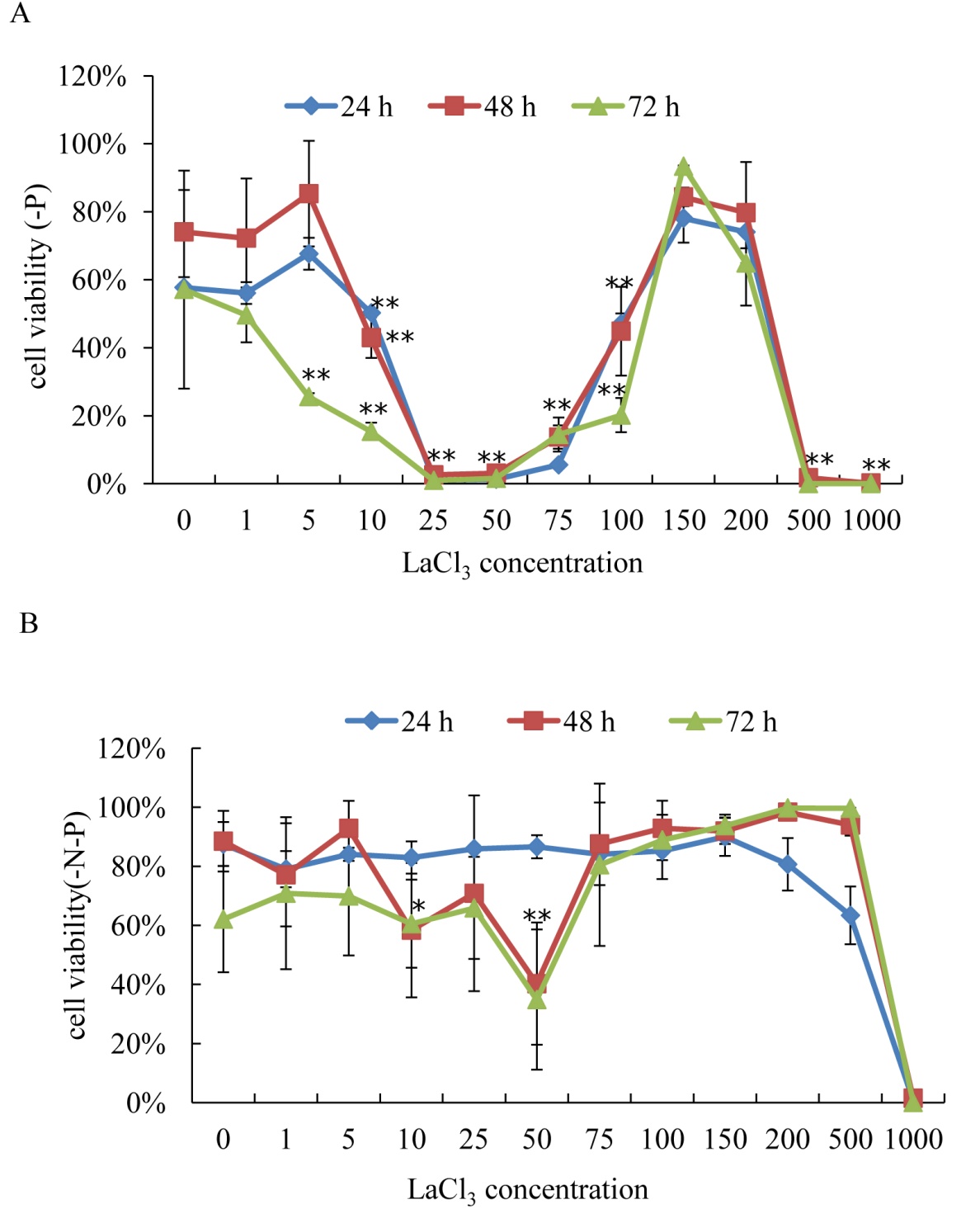


Fig S3 Cellular viability of the algal cells treated with various concentration of LaCl_3_ under P (A) and P & N (B) starvation.

*C. reinhardtii* CC425 cells were inoculated into 100 mL Erlenmeyer flasks containing 50 mL of TAP media for 3 days to reach a growth plateau and collected by centrifugation 3000g. After washed twice with sterile water, the cultured cells were inoculated into HSM media lacking P (-P), and N & P (-N-P) with various concentrations of LaCl_3_ (0, 1, 5, 10, 25, 50, 75, 100, 150, 200, 500 and 1000 μM). “0 μM” LaCl3 treated cells were used as control. Samples were collected at 24 h, 48 h and 72 h timepoints. The algal cells were directly stained with 0.01% (w/v) fluorescein diacetate (FDA) (final concentration) for 2 min, and then the cell number was counted by a flow cytometry CyFlow®Cube 6. A total of 10000 cells were counted per sample. Cellular viability are shown as the precentages of FDA-stained live cells. ANOVA (combined with Duncan’s multiple range tests) was employed for statistical analysis, and the experiment was repeated three times. Significance is indicated as *p < 0.05, and **p < 0.01

Fig S4 The cell density of Maa7- and RNAi strains grown in HSM agar medium contain 0.5 mM ammonium for 12 days.

The agal cells were picked into 200 μL steriled water.
